# Transactions of the Chicago Society of Physicians and Surgeons

**Published:** 1875-02-15

**Authors:** 


### Society Reports.

### TRANSACTIONS OF THE CHICAGO SOCIETY OF PHYSICIANS AND SURGEONS.

### REGULAR MEETING, FEBRUARY 8TH, 1875.

THE President, Dr. John Bartlett, called the meeting to order.

Dr. C. W. Burrell read the following report of a case of

SUPPURATIVE PLEURITIS.

While visiting for the Relief and Aid Society for the purpose of diagnosing cases, claiming aid because of sickness, I was requested February 7th, 1874, to examine the subject of these notes.

Annie B——, a girl 16 years of age; she is of German parentage, and a member of a healthy, robust family. I learned from herself and mother the following history: On Dec. 13th,

1873, after being exposed by washing outside steps, she experienced a chill, which was followed by fever and very severe pain in the left side. The physician who was called, pronounced her trouble pleuro-pneumonia. On the second or third day, the characteristic expectoration of pneumonia appeared. The pain and fever subsided, but the prostration and cough continued, the expectoration gradually ceasing. The physician who first saw her was still in attendance, and supposed the pneumonia had become chronic. She was taking tonics; was emaciated and very weak; found her in bed; was coughing often, but expectorating but little.

Examination, Saturday evening, February 7th, revealed: 1st, by inspection, perceptible bulging of left side; intercostal spaces full on left side; depressed on right; left side measuring 1½ inches more than right; no perceptible motion of left side. 2d, by percussion, flatness over entire left lung, excepting a strip about one inch along mesian line, both anteriorly and posteriorly, and in subclavicular region, where dullness was pretty well marked; no succussion could be elicited. 3d, by auscultation, there were no rales, no bronchial breathing, no bronchophony, no vocal fremitus; in fact, there was absolute quiet on the left side; increased vesicular murmur on right.

Notwithstanding the history of pneumonia, and diagnosis of the attending physician of chronic pneumonia, I reported the case to the Society as one of Suppurative Pleuritis, and advised she be sent to an hospital, the surroundings of her home and circumstances of her parents, not permitting of proper care and treatment there. Gave an opiate to allay the cough; and as I was passing the next day, called to see her.

A change had occurred. During a severe fit of coughing, expectoration, purulent and very profuse, began. An opening into the bronchial tubes had been effected. The abundant expectoration continued; she was sent to the hospital Tuesday morning, February 10th. There she was carefully examined, and the disease diagnosed as chronic pneumonia. Prognosis decidedly unfavorable. Tonics — cod-liver oil, quin. sul. and iron—were given. The expectoration continued very profuse. She gained for a time only. After remaining in hospital for fourteen weeks without further relief, she returned home, and her mother requested me to see her, which I did, June 20th, more than six months after the origin of the trouble.

The cough was almost incessant, permitting of but little rest day or night. She was very much emaciated and nearly exhausted; could scarcely walk across the floor; pulse 120; no appetite; expectorating muco-purulent matter very freely. Examination revealed the same condition of things as did my first. The left side measured 1½ inches more at base, and ¾ inch more under arms, than the right side. Flatness on percussion. Almost absolute silence on auscultation. Now that she could be under my control, I determined to explore the pleural cavity, which I did, assisted by Dr. Kelsey, June 22d. By means of a Codman & Shurtleff aspirator, using a No. 2 needle, 78 ounces of creamy pus were removed. During the evacuation, which required an hour and a quarter, bronchial breathing, together with a tinkling sound, were prominent. Toward the close, air passed through the needle from the chest. The lung did not expand very well. A few doses of arom. spts. ammon. were given before and during the operation, and the patient bore it well, expressing herself relieved. Ordered cod-liver oil and n. m. acid. She rested and slept well. Had no cough for forty hours. Appetite and strength improved. On June 24th, by shaking the body, she could produce a succussion sound, which was audible at some distance. The cavity rapidly refilled, the expectoration again became profuse; the cough began to deprive her of a sufficient amount of sleep. On July 1st, being assisted by Drs. Lester, Curtis and Kelsey, I repeated the operation. The pus was thinner and darker colored; 68 ounces were removed; the operation well borne, and she improved rapidly. Oil and acid continued. July 3, she rode down town, and on the fourth, she walked ten blocks.

The appetite became almost ravenous.

During the night of July 5th, she had cholera morbus very severely.

July 8th. — Nearly regained her strength. Cavity filled to inferior angle of scapula. No cough. Pulse 98. July 10th.—Doing well. Appetite and strength returned. Coughing and expectorating sero-purulent matter. Pulse 88.

The operation was repeated on July 13th, 20 ounces of thin pus being withdrawn. She improved rapidly in weight and strength. The hair, which had fallen out, again began to grow. The oil and acid were continued. On July 21, the cavity had filled to a point an inch below inferior angle of scapula. No cough. Pulse 76. Appetite good. Sleeps well all night.

In my notes under date of August 4th, I find the following: No cough. Strength increasing. Assists in work about the house. Owing to neglect of patient in coming to my office, the operation has been delayed. As she gets better, she dreads the operation. The cavity is filled to a point higher than that at which expectoration began before. This would indicate a closure of the opening into the bronchial tubes. The upper part of lung doing good service.

Sept. 25th.—So much improved that she has neglected to come to me. Have not examined her since last date.

Sept. 30th. — Feels quite well. There is retraction of left side. The lung is acting pretty well throughout its upper half. Flatness and silence over lower portion. Operated. This time failed to get any pus.

From this time on, her health was so good, that further interference was not deemed best. She has steadily improved. Has become as strong, and more fleshy than ever in her life. Has no inconvenience whatever from that side.

The peculiarities and interesting points of this case are, the different diagnoses, the mode of operating, the duration of the disease, its course, and the rapid and complete recovery.

Dr. Hyde next gave a verbal report of a case of delivery of a living child, at seven months, after artificial contraction of the vagina, for the relief of procidentia uteri. The case is not completed, but will be subsequently reported in full. The Moleswortn dilator was used in order to enlarge a cicatrical contraction of the vagina, and premature labor was induced by its presence only.

Dr. Sawyer remarked that he had made successful use of the Molesworth dilator, for the arrest of an obstinate epistaxis, and had also made use of it in two cases of stricture of the rectum. In the first, a case of syphilitic stricture, the relief was only temporary; in the second case, a perfect cure was effected. At his suggestion, Dr. Bevan had also used the dilator in a similar case, with entire success.

Returning to the question of the best means of dilating the os, Dr. Sawyer remarked, that Dr. St. Clair, in an article in the Boston *Journal*, proposes to use the hand in all cases. One strong objection to the introduction of the hand before labor for the dilatation of the os was, the severe pain caused by the operation, so that it was only justifiable when the patient was placed under the influence of an anæsthetic. The uneven hand, Dr. Sawyer considered, was under any circumstances, inferior for the purpose, to the Brows or Molesworth dilators.

In regard to Crede's method of squeezing out the placenta, of which mention was made at the previous meeting, in the discussion of Dr. Merriman's paper, Dr. Sawyer said, that in his early experience in dispensary practice, he had employed the method in twelve cases, and had as a result, prolapsus uteri in eight of the cases.

Dr. Merriman, from the committee on clinical reports, next reported verbally, two cases of

GASTRO-INTESTINAL HÆMORRHAGE IN INFANTS.

The first case, a strong, vigorous child, thirty-six hours old, was attacked suddenly, and without apparent cause, with a vomiting and purging of blood. On the arrival of the doctor, the discharges had ceased spontaneously, but the child presented an extremely pale, exsanguine look. A mixture of fl. ext. ergot and tinct. ferri. chlor. was prescribed, and the child had no further trouble.

A second, somewhat similar case, occurred in a child forty-eight hours old. Blood was vomited in small quantity, two or three times, but no purging. Both were plethoric infants, had appeared perfectly well otherwise, and had begun to nurse before the discharges took place. Some of the authorities assign the too quick tying of the cord as the cause of these hæmorrhages. This cause could not have operated in either of these cases, however.

Dr. Merriman next referred to a case of severe constitutional syphilis, under his care, in Mercy Hospital, which, after failing to improve under any variety of syphilitic treatment, rapidly regained health upon the substitution of tonics, cod-liver oil, etc., for the mercurials and alteratives which had been given. Later, as general strength improved, the iodides were again resorted to with benefit.

In conclusion, the doctor referred to a case of puerperal fever, that had just died under his care; mental despondency and worry, he believed, to have been the chief cause of the supervention of the fever. A majority of the cases of this disease, he thought, were directly traceable to some family trouble, or cause producing mental depression.

Dr. Hyde referred to the use of oleate of mercury, externally, in the treatment of syphilis. It was free from many of the objections attached to the ordinary internal administration of mercury, and produced no general disturbance, or debilitating effect, upon the system. The iodides could be administered internally at the same time.

Discussion followed by several members as to the connection supposed to exist between puerperal fever and erysipelas; and also, as to whether a physician was justified in attending cases of labor soon after having performed a *post-mortem*, or having been engaged in dissecting.

By vote of the Society, Dr. Bartlett was requested to prepare a report on this subject for presentation to the Society.

On motion, the Society then adjourned.

